# Supplementary material for: Impact of Electrolyte Incorporation in Anodized Niobium on Its Resistive Switching
Source: Nanomaterials (Basel). 2022 Feb 28;12(5):813. doi: 10.3390/nano12050813 (PMC8912554; doi:10.3390/nano12050813)
Supplement: Supplementary file 1 [file nanomaterials-12-00813-s001.zip › nanomaterials-1586177-supplementary.pdf]

Impact of electrolyte incorporation in anodized niobium on its resistive switching

Ivana Zrinski[1], Marvin Löfler[1], Janez Zavašnik[2], Claudia Cancellieri[3], Lars P.H. Jeurgens[3], Achim Walter Hassel[1] and Andrei Ionut Mardare[1,*]

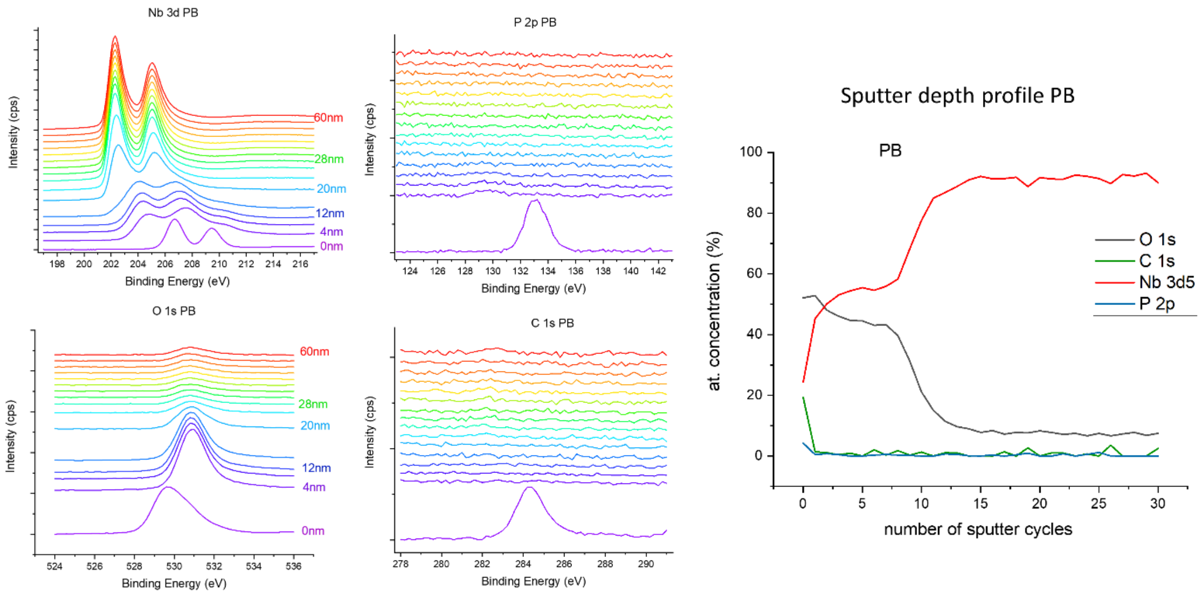

Figure S1: XPS depth sputtering profiles for anodic Nb memristors grown in phosphate buffer.

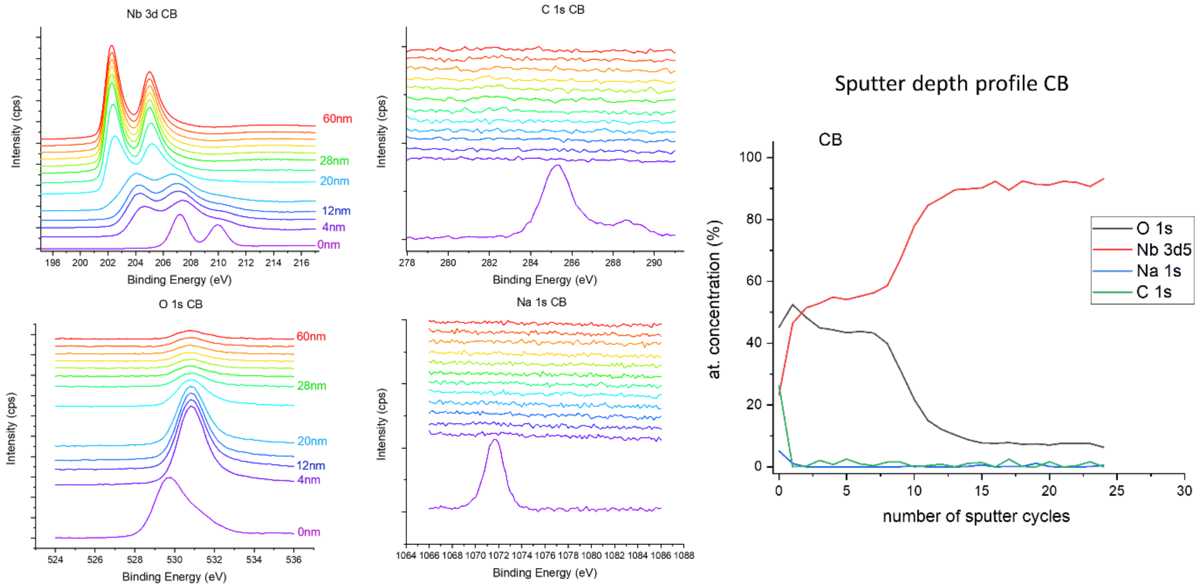

Figure S2: XPS depth sputtering profiles for anodic Nb memristors grown in citrate buffer.

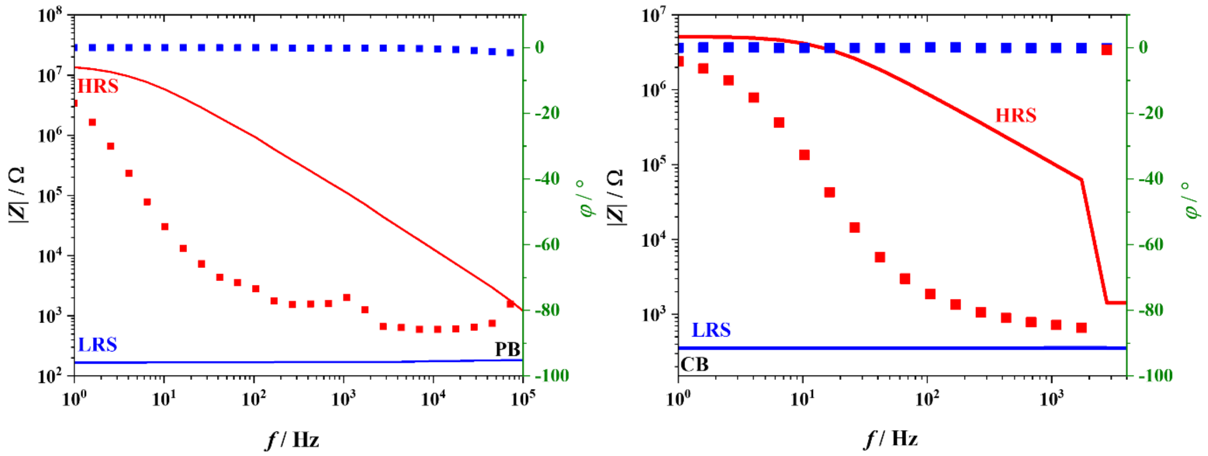

Figure S3: Impedance spectroscopy Bode plots of memristors grown in different electrolytes switched in LRS and HRS showing their impedance (full lines) and phase shift (dotted lines) dependence on frequency.
